# Getting Started in Computational Immunology

**DOI:** 10.1371/journal.pcbi.1000128

**Published:** 2008-08-29

**Authors:** Steven H. Kleinstein

**Affiliations:** Interdepartmental Program in Computational Biology and Bioinformatics, and Department of Pathology, Yale University School of Medicine, New Haven, Connecticut, United States of America; Princeton University, United States of America

The immune system acts across multiple scales involving complex interactions and feedback, from somatic modifications of DNA to the systemic inflammatory reaction. Computational modeling provides a framework to integrate observational data collected from multiple modes of experimentation and insight into the immune response in health and disease. This Message attempts to illustrate how different computational methods have been integrated with experimental observations to study an immunological question from multiple perspectives by focusing on a very particular, though fundamental, component of adaptive immunity: B cells and affinity maturation (Figure 1). B cells bind foreign antigens through their Immunoglobulin (Ig) receptor. Affinity maturation is the process by which B cell receptors that initially bind antigen with low affinity are modified through cycles of somatic mutation and affinity-dependent selection to produce high-affinity memory and plasma cells. How this process can reliably generate orders of magnitude increases in affinity over a period of weeks is one of the many questions where computational modeling has made important contributions (for example, the cyclic re-entry model [1]). Yet, even the seemingly straightforward matter of detecting antigen-driven selection remains controversial, and such fundamental questions as whether increased proliferation or decreased death drives the preferential expansion of higher-affinity B cell mutants remain unanswered. A good biological introduction to the immune system is available on the NIH website [2], while more detailed information can be found in any number of textbooks [3]. An animation by Julian Kirk-Elleker provides a visual introduction to the affinity maturation process (http://web.mac.com/patrickwlee/Antibody-affinity_maturation/Movie.html). The kinds of computational techniques described here have been widely applied in other areas of immunology, including the innate response [4],[5], viral dynamics [6], and immune memory [7]. A classic introduction to computational immunology geared to the more mathematically inclined was written by Perelson and Weisbuch [8]. The rapidly expanding area of immunoinformatics was covered in a recent issue of *PLoS Computational Biology* [9], and several other applications were explored in a 2007 volume of *Immunological Reviews* (216) devoted to quantitative modeling of immune responses.

**Figure 1:**
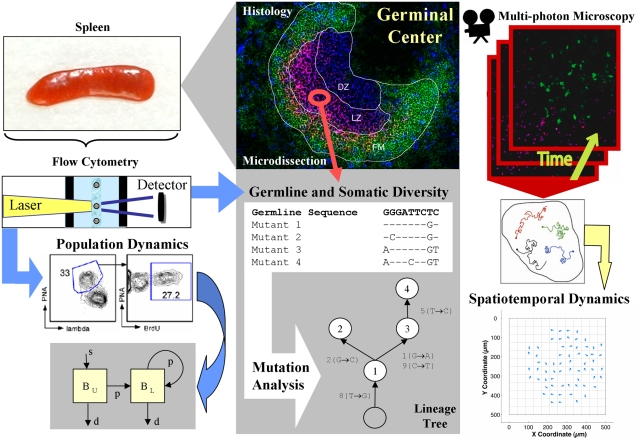
A wide range of experimental techniques are used in combination with computational modeling to probe the process of affinity maturation at multiple scales (from DNA to tissue). Population dynamics of splenic germinal center B cells is probed by quantifying labeled cells over time with flow cytometry (left panes). Microdissection of cells from tissue sections combined with sequencing of the Ig receptor provides information on germline receptor usage and somatic hypermutation (center panes). Histology is supplemented with intravital multi-photon microscopy to visualize and quantify spatiotemporal dynamics (right panes).

## Germline and Somatic Diversity

The adaptive immune system operates by clonal selection. A preformed repertoire of diverse Ig receptors for antigen is clonally distributed among a finite but large number of B cells. These receptors are generated by a somatic recombination process that brings together a number of interchangeable gene segments present in the DNA. Recombination signals (RSs) associated with each segment help determine the efficiency of segment pairing, but high variability both across and within species has made experiments difficult to interpret. Computational models have been used effectively to exploit the correlation structure of known RSs to predict recombination efficiency and to recognize new RSs [10]. Hypotheses concerning gene segment usage (e.g., random versus sequential) have also been investigated using probabilistic models to simulate the distribution of cells with different rearrangements [11]. Along with investigating the “how” of Ig rearrangement, computational modeling has been used to explore why such diversity is necessary [12].

Foreign antigens are recognized by individual B cells that happen to have receptors that bind, with the threshold for activation being set low, since in general these chance “fits” between receptor and pathogen will have weak interactions. During the course of an immune response, Ig receptors that initially bind antigen with low affinity are modified through cycles of somatic mutation and affinity-dependent selection to produce high-affinity memory and plasma cells. Somatic mutation is a process unique to B cells responding to antigen that results in a mutation rate that is 7–8 orders of magnitude above normal background (and thus often referred to as hypermutation). Identifying somatic mutations in experimentally derived Ig receptor sequences is critical to understanding this process, but can be challenging since the germline sequence for individual B cells is chosen stochastically during cell maturation in the bone marrow and thus is not known a priori. Imprecision in the recombination process, and the action of various enzymes that can add or delete nucleotides during rearrangement, further compounds this problem. Hidden Markov models and other computational approaches have been instrumental to predict germline sequences, including the most likely combination of gene segments involved [13],[14].

Analyzing the interaction between somatic hypermutation and germline codon usage in the Ig receptor has provided insight into strategies used by the immune system to adapt to pathogenic challenge. In general, more mutable codons are used in the complementary determining regions (CDRs), where most contact residues for antigen binding are found, and less so in framework (FW) regions, which provide the structural backbone of the receptor [15]. This suggests that Ig receptors have evolved to focus mutations to maximize potential benefit and minimize the possibility of producing non-functional receptors, although not all isotypes behave the same way [16]. A critical resource for these kinds of studies is the IMGT database (http://imgt.cines.fr), which contains a wealth of sequence information, including the germline Ig genes of several species and links to analysis tools.

## Mutation Analysis

The mutation patterns in experimentally derived Ig sequences provide a kind of fossil record for the affinity maturation process, and can furnish important evidence of antigen-driven selection. The most common tests for selection compare the observed frequency of replacement mutations to their expected frequency under the null hypothesis of no selection. Elevated frequencies indicate positive selection, while decreased levels indicate negative selection with significance determined by a binomial test [17],[18]. Such inferences depend on the difficult task of accurately defining the features of a “random” mutation process. A main problem is that somatic hypermutation, while stochastic, displays intrinsic sequence-specific biases that can give the appearance of selection. This has led some to suggest that such methods cannot be used as reliable indicators of antigen-driven selection [19], while our own work shows that more comprehensive models along with better statistics can be used to detect selection in vivo with high specificity [20].

Low sensitivity is another problem that plagues methods for detecting selection. Additional information may be extracted from B cell lineage trees (also called clonal trees), which depict the relationships between groups of B cells that share a common ancestor (often generated from microdissection experiments). Unlike the case for phylogenetic trees, the relatively small number of mutations and sequences means there are often few ambiguities in creating these trees. Monte Carlo simulation approaches have been used to link the topological properties of B cell lineage trees to underlying biological processes such as somatic hypermutation [21] and selection [22]. Inferences based on lineage tree properties are challenging since many different biological processes can produce similar changes in tree shape, and direct tests for selection based on these properties have yet to be developed for the immune response.

## Population Dynamics

Affinity maturation involves extensive proliferation and death. Accurate rate measurements for these processes can help determine their relative contribution to the preferential expansion of higher-affinity B cell mutants. Dividing cells can be labeled using bromodeoxyuridine (BrdU), a thymidine analog that gets incorporated into DNA during S phase. The fraction of labeled cells is tracked during BrdU administration and following withdrawal using flow cytometry. To interpret these data, Bonhoeffer et al. [23] proposed a simple model that assumes a single B cell population that proliferates at rate *p* and undergoes apoptosis at rate *d*. To model BrdU labeling, this population is split into unlabeled (B_U_) and labeled (B_L_) subsets:
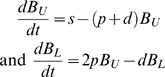
where we have assumed an unlabeled source of cells (*s*) and 100% labeling efficiency.

Similar kinds of population dynamic models have been developed to help interpret experiments using the cell dye carboxyfluoroscein succinimidyl ester (CFSE) [24]. In this case, division results in a halving of the signal intensity so that each measurement provides information on the number of divisions undergone by individual cells since labeling. Proliferation and death rates are estimated by parameter optimization producing the best (e.g., least-squares) fit of the model with experimental data. Confidence intervals are often determined using bootstrapping. However, the simplest models, such as presented above, often do not provide good fits, and significant controversy still exists as to the proper model to use for a particular situation [25],[26].

The biological mechanisms underlying the preferential expansion of rare higher-affinity B cell mutants are largely unknown. Population dynamic models including somatic hypermutation and selection can be used to explore the consequences of different hypotheses. Indeed, such modeling played an important role in suggesting that a process involving cyclic re-entry was necessary to achieve efficient affinity maturation [1], and showed how it could be mapped onto the micro-architecture of germinal centers (the sites of affinity maturation that form in the secondary lymphoid organs during immune responses) [27]. Other studies have investigated different selection mechanisms, including competition for space [28],[29]. The predicted efficiency of affinity maturation can depend on the underlying model of the affinity landscape. While some models use decision trees to simulate the mutation process [20],[30], other frameworks have also been developed to capture statistical properties of somatic hypermutation and affinity maturation [1],[31],[32]. The estimation of unknown parameters is another important component of these studies, and it is common to choose values that maximize affinity maturation (under the assumption that evolution has optimized this process). However, quantitative modeling of specific responses has predicted that many cells with affinity-increasing mutations are not expanded as would be expected for optimal affinity maturation [33],[34]. Indeed, there is still ongoing discussion about why B cells mutate their Ig receptors at all [35], an area where computational modeling should be able to make important contributions.

## Spatiotemporal Dynamics

The spatial structure of the germinal center is thought to play an important role in affinity maturation, and many models include multiple compartments. However, it has only recently become possible to visualize the spatiotemporal dynamics of immune responses in vivo using “intravital multi-photon microscopy,” which allows tracking of individual cells in the lymph nodes and germinal centers [36]. While much initial work has focused on statistical analysis of different cell populations (e.g., comparisons of velocity and displacement rates), and addressing the question of whether cell movement is random or directed, more detailed computational modeling will play a key role in understanding these complex datasets. Spatially explicit simulations have already led to the important insight that some migration behaviors, such as directed motion on a short timescale and random motion on a longer timescale, may result simply from the crowded microenvironment of the lymph nodes [37]. Other studies have started to integrate data from several experiments to link models of affinity maturation with migration patterns, raising questions about whether the widely accepted cyclic re-entry model can be consistent with the observed efficiency of affinity maturation [38]. Integrating observations from different modes of experimentation (Figure 1) continues to be a challenge.

## Discussion

Modeling and computational approaches have been widely applied to problems in immunology, and are finding increasing applications as experiments become more quantitative and seek to extract information on kinetics. Virtually all of the top immunology journals now publish papers with significant computational components, which was not the case just a few years ago. In some ways, this success presents a challenge for those looking to get started in the field. Leading computational immunology research groups often publish their work in domain-specific experimental journals and present at biology conferences, so finding and following state-of-the-art research requires tracking several journals and becoming familiar with many different areas of biology.
